# Household preferences for reducing greenhouse gas emissions in four European high-income countries: Does health information matter? A mixed-methods study protocol

**DOI:** 10.1186/s12889-017-4604-1

**Published:** 2017-08-01

**Authors:** Alina Herrmann, Helen Fischer, Dorothee Amelung, Dorian Litvine, Carlo Aall, Camilla Andersson, Marta Baltruszewicz, Carine Barbier, Sébastien Bruyère, Françoise Bénévise, Ghislain Dubois, Valérie R. Louis, Maria Nilsson, Karen Richardsen Moberg, Bore Sköld, Rainer Sauerborn

**Affiliations:** 1 Institute of Public Health, Heidelberg University Hospital, Im Neuenheimer Feld 130.3, 69120 Heidelberg, Germany; 2 Institute of Psychology, Heidelberg University, Heidelberg, Germany; 3 TEC-Conseil, Marseille, France; 4 Vestlandforsking, Sogndal, Norway; 5 Department of Public Health and Clinical Medicine, Epidemiology and Global Health, Umeå University, Umeå, Sweden; 6 Centre International de Recherche sur l’Environnement et le Developpement (CIRED), Nogent, France

**Keywords:** Climate change, Health co-benefits, Mitigation, household preferences, Mixed-methods, Policy

## Abstract

**Background:**

It is now universally acknowledged that climate change constitutes a major threat to human health. At the same time, some of the measures to reduce greenhouse gas emissions, so-called climate change mitigation measures, have significant health co-benefits (e.g., walking or cycling more; eating less meat). The goal of limiting global warming to 1,5° Celsius set by the Conference of the Parties to the United Nations Framework Convention on Climate Change in Paris in 2015 can only be reached if all stakeholders, including households, take actions to mitigate climate change. Results on whether framing mitigation measures in terms of their health co-benefits increases the likelihood of their implementation are inconsistent. The present study protocol describes the transdisciplinary project HOPE (HOuseholds’ Preferences for reducing greenhouse gas emissions in four European high-income countries) that investigates the role of health co-benefits in households’ decision making on climate change mitigation measures in urban households in France, Germany, Norway and Sweden.

**Methods:**

HOPE employs a mixed-methods approach combining status-quo carbon footprint assessments, simulations of the reduction of households’ carbon footprints, and qualitative in-depth interviews with a subgroup of households. Furthermore, a policy analysis of current household oriented climate policies is conducted. In the simulation of the reduction of households’ carbon footprints, half of the households are provided with information on health co-benefits of climate change mitigation measures, the other half is not. Households’ willingness to implement the measures is assessed and compared in between-group analyses of variance.

**Discussion:**

This is one of the first comprehensive mixed-methods approaches to investigate which mitigation measures households are most willing to implement in order to reach the 1,5° target set by the Paris Agreement, and whether health co-benefits can serve as a motivator for households to implement these measures. The comparison of the empirical data with current climate policies will provide knowledge for tailoring effective climate change mitigation and health policies.

**Electronic supplementary material:**

The online version of this article (doi:10.1186/s12889-017-4604-1) contains supplementary material, which is available to authorized users.

## Background

It is now universally acknowledged that climate change constitutes a major threat to human health [1]. Although its impacts can in principle be positive and negative for health, most empirical studies suggest that negative health impacts will outweigh positive ones [2–4]. Climate change can intensify the spread of communicable diseases, such as malaria or diarrheal diseases, by improving environmental conditions for disease vectors [5–7]. Moreover, climate change can increase morbidity and mortality of non-communicable diseases, such as cardiovascular or respiratory diseases, for instance by favoring extreme heat and longer duration of the pollen season [6, 8, 9]. Indirectly, climate change can impact health by bringing about social or ecological disruptions, for instance through extreme weather events or sea level rise, potentially leading to population displacement or malnutrition and reducing global food security, at least on a regional scale [4, 10–12].

At the same time, some of the measures to reduce greenhouse gas emissions, so-called climate change mitigation measures, have significant health co-benefits [13–15]. Health co-benefits are “health gains from strategies that are directed primarily at climate change, and mitigation of climate change” [1]. On the one hand health co-benefits can arise from changes in lifestyles, which are more healthy and more climate-friendly. For instance, this is the case, when someone decides to eat less meat, which reduces this person’s risk of cardiovascular mortality and some cancers and at the same time reduces emissions from raising cattle [16]. Another example is the personal choice of biking or walking to work (so-called ‘active transport’) instead of using a private car, which increases cardiovascular health of that individual independent of mitigation actions taken by other stakeholders [17]. We call these effects direct, because they have a measurable direct impact on the individual taking action.

**Fig. 1:**
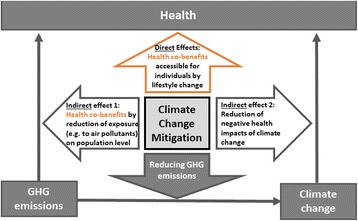
The effects of climate change mitigation on health (*health co-benefits* and others). Climate change mitigation measures adopted by an individual can directly affect this individual’s health, if the health effect is accessible by personal choice (e.g. *health co-benefits* of reduced cardiovascular health risks by biking to work or eating less meat). Climate change mitigation measures adopted by many individuals can indirectly effect health on population level (e.g. *health co-benefits* of reducing greenhouse gas emissions and therefore air pollution, reducing e.g. respiratory and cardiovascular morbidity and mortality). Moreover, successfully mitigating climate change can reduce the negative health effects of climate change itself. (Please note, that this effect on health is no *health co-benefit)*. Households in the HOPE study are only presented with information about direct health co-benefits accessible for individuals by personal choice (*upper orange arrow*)

On the other hand, there are health co-benefits which result from the reduced exposure to substances which are climate active and have negative health impacts, for instance air pollutants. Climate policies which are aimed at reducing greenhouse gas emission often reduce air pollution at the same time, which leads to the reduction of e.g. cardiovascular and respiratory morbidity and mortality on population level [18]. However, as these effects only occur if there is collective action and are not directly accessible by a single individual, we consider these effects as indirect. Another indirect way in that climate change mitigation can affect health, is that mitigation reduces the negative health effects of climate change, as described above. However, these kinds of effects are not considered as health co-benefits. Figure 1 illustrates the effects of climate change mitigation on health as described above.

In order to mitigate climate change, the European Union (EU) put forward a greenhouse gas (GHG) emissions reduction target of at least 40% by 2030 compared to 1990 emission levels [19]. However, even deeper cuts are necessary to attain the currently targeted 1.5 °C warming limit of the Paris Agreement in 2015 at the Conference of the Parties (COP) to the United Nations Framework Convention on Climate Change (UNFCCC) [20]. To reach strict emission targets a societal transformation with profound changes in production, consumption and lifestyles, is needed [21]. Nevertheless, climate policies on private consumption and lifestyles are relatively rare, even though households are key actors in global GHG-mitigation [22–24]. Today, up to 72% of global emissions are related to household consumption in high income countries [25].

Yet to engage households in climate change mitigation was shown to be difficult [26, 27]. Sauerborn et al. and Myers et al. pointed out that health was a potential motivator to adopt mitigation action [28, 29]. This is because health provides a positive message as a motivator, saying that "what is good for the climate is good for your own health". Empirical research in the fields of cognitive psychology and public health promotion demonstrates that messages framed in terms of possible gains are more effective in achieving behavior change than messages framed in terms of the prevention of losses [30]. More specifically, in the climate change domain, one study showed that framing climate change in terms of the achievement of a more caring society or technological progress, enhanced pro-environmental action intentions in climate deniers compared to framing it in terms of risk avoidance [31]. In another study involving a large community sample, frames around positive values and visions were more effective in promoting mitigation behavior intentions than frames around personal sacrifices [32]. Yet, negative scenarios, sometimes called “doom-and-gloom scenarios”, abound in communications of climate change to the public.

Research on whether health as a possible gain from mitigation measures motivates people to adopt individual mitigation measures is still scarce and controversial. Many studies have shown that a health frame can enhance individual engagement and support for climate change mitigation [28, 33–35]. Furthermore, research on what motivates people to buy organic food, shows that perceived health benefits of organic feed are more important for consumers than environmental benefits. This is especially interesting, because the scientific proof for health benefits (on the individual consumer) is far less supported by scientific studies than that of environmental benefits of organic food [36]. On the other hand, a recent large study has shown that simply reframing GHG mitigation efforts in terms of their health co-benefits is unlikely to increase individuals’ support for climate change mitigation [37].

## Methods

### Study objectives

The present study protocol describes a transdisciplinary research project investigating how information on health co-benefits influences household’s choices of climate change mitigation actions in urban households in France, Germany, Norway, and Sweden. The project at large investigates HOuseholds’ Preferences for reducing greenhouse gas emissions in four European high-income countries (HOPE) with researchers from public health research, psychology, political science, sustainability studies, environmental research and economy. HOPE concentrates on health co-benefits, which are directly accessible to individuals, such as reducing meat consumption or making use of active means of transport (see upper arrow in Fig. 1). This procedure stands in contrast to general and impersonal health framings of mitigation efforts which have previously been tested in research yielding mixed results [37].

To gain a deeper understanding of the question of health as a motivator to adopt climate change mitigation measures in households, HOPE will use a mixed-methods approach combining quantitative and qualitative research methodologies. HOPE investigates the following broad research objectives:

1. To investigate households’ motivations and barriers to adopt climate change mitigation measures, especially with regard to the role of direct health co-benefits in households’ preferences for those measures.
2. To investigate which mitigation measures households in European high-income countries prefer to adopt under the goal of reducing emissions by 50% by 2030 addressing the 1.5 °C goal.
3. To estimate the GHG emission reduction potential and private economic impact of the preferred and non-preferred mitigation measures as well as respective costs and savings for households.
4. Investigate the link between climate policies and household preferences as well as the potential implications of our findings for policy makers on local, national and EU-level.

In this paper we focus in particular on presenting the part of the HOPE project that deals with the aspect of health co-benefits. We expect that providing households with information on the individual health co-benefits of adopting mitigation measures will increase their preference to select these measures.

### Mixed-methods design

**Fig. 2:**
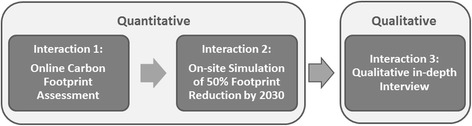
Explanatory mixed-method design of the HOPE-Study. The explanatory mixed-methods design comprises three steps (=Interaction 1–3). The first two steps use quantitative methods, the third step uses qualitative methods

HOPE applies an explanatory mixed-methods design comprising three steps of data collection termed household *Interactions*. Data collection of Interactions 1 and 2 are done in parallel, and are quantitative. Interaction 3 is done approximately 3 months after completion of Interactions 1 and 2 to build upon their results, and will be purely qualitative (see Fig. 2). This kind of mixed-methods design is called explanatory, because the qualitative part of the research is designed to further explain results from the quantitative part [38]. Each participating household is asked to select one household representative to participate in the study on behalf of the whole household. This was decided to ensure similar conditions of data collection for each household excluding group dynamics. The household is free to decide who should be the representative as long as he or she is aged over 18 years of age. Interactions 1 and 2 were pre-tested in a pilot study in all partner countries in December 2015 and January 2016. Data collection was accomplished from June 2016 to June 2017.

### Three steps of data collection

#### Interaction 1: Online carbon footprint assessment

##### Scientific basis

The carbon footprint is a core concept in consumption related climate research. We define a household’s carbon footprint to include all GHGs measured as CO_2_-equivalents in tons, which are caused directly or indirectly by the household’s activity in 1 year [39].

##### Procedure

In Interaction 1, we assess each household’s carbon footprint. Households fill in an online questionnaire to attain the necessary information. For households who are not willing or able to fill in the questionnaire online, a paper version is provided, an assistant helps the household fill in the questionnaire, or a hotline is available (depending on country) that helps to clarify questions. Participants first provide socio-economic data of the household such as age of household members, education of the household representative, or household income. The main part of the questionnaire is concerned with detailed household consumption patterns and spendings in the four areas of *housing*, *mobility*, *food* and *other consumption*.

The data are then transferred into a footprint calculation and simulation tool (FCS-tool), specifically developed for the HOPE study. The FCS-tool calculates the initial carbon footprint of the household and simulates the emission reduction, cost development and health effects of households’ mitigation choices. The exact functioning of the tool is described elsewhere (Dubois et al., forthcoming). An overview of all variables collected in HOPE Interaction 1 and 2 can be found in the Additional file 1.

#### Interaction 2: On-site simulation of 50% carbon footprint reduction by 2030

##### Scientific basis

The core of HOPE, interaction 2, is an on-site simulation, evaluating household’s preferences and choices for a set of personalized mitigation actions. The households’ preferences are represented through the statement of the household’s willingness to perform the actions, measured by ratings and choices. In this sense, HOPE applies principles of Contingent Choice Surveys, a method commonly used to elicit stated preferences in environmental issues [40]. Furthermore, interaction 2 applies elements of the method of serious gaming. Serious games apply game principles, such as a narrative or rules, for non-entertainment purposes, such as learning or research [41]. They are a well-established tool to engage study participants into participatory, integrated assessments of complex socio-technical policy problems [42, 43]. Last, in interaction 2 HOPE applies an experimental design in order to test the effect of giving information on direct health co-benefits of mitigation actions versus not giving this information.

##### Material

**Fig. 3:**
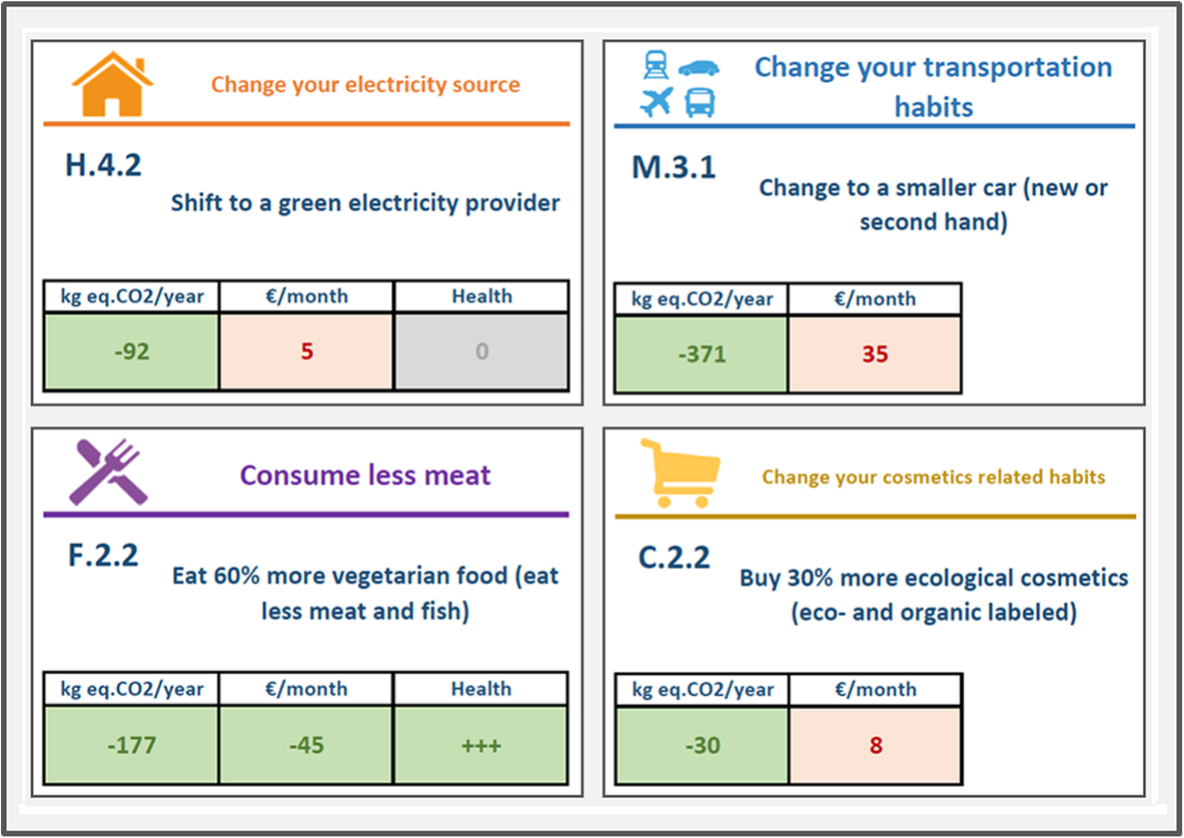
Action cards on mitigation options in Interaction 2. Examples of action cards (translated into English). Action cards are color-coded for the category the mitigation option belongs to (housing, food, mobility, or other consumption – see also symbols in the upper left corner). In the lower part of the action card two or three boxes with additional information are presented to the household, depending on the experimental group the household belongs to health (health information vs. no health information). Each household receives information on reduction of CO_2_ emissions and money spent or saved associated with implementing the mitigation option (*left and middle box*). Half of the households additionally receive information on how their health is affected when implementing the measure (*right box*)

Interaction 2 is centered around 65 possible household mitigation options (e.g. using public transport more; eating less meat; reducing room temperature by 1 °C). Each possible mitigation option is displayed on one *action card* (Fig. 3), and falls within one of the four sectors housing, food, mobility, and other consumption (see symbols in Fig. 3). In addition to the mitigation action, each action card displays additional information on the amount of CO_2_ emissions that this action can save and the monetary costs or savings of this action. Emission information displays the emission savings associated with implementing the measure (in kg of CO_2_-e saved per year), the cost information displays the costs or savings associated with implementing the measure (in Euros/Kronor per month).

Half of the households additionally receive information on the health effect of this measure. Health information displays the health benefits or harms associated with implementing the measure. The health information is calculated on a generic level based on the concept of Quality Adjusted Life Years (QALYs) as described in chapter 2.5 below. Since households cannot be expected to be familiar with the concept of QALYS, health effects are expressed as ‘+’ to indicate a small positive effect, ‘++’ to indicate a moderate positive effect, ‘+++’ to indicate a substantial positive effect and ‘-‘to indicate a negative effect. We provide only half of the households with health information in order to create an experimental design with a treatment group receiving health information and a control group not receiving health information. The health information is deliberately given in a discrete way aiming for the measurement of an experimental effect in a realistic and complex simulation, which reflects real life decisions.

The mitigation measures are specifically tailored to individual households in two ways. First, measures that do not apply to a specific household as known from Interaction 1 are crossed out on the action card. For example, reducing meat consumption is crossed out for vegetarian households. These cards are still offered to the participant to double-check, but are then coded as non-applicable in the FCS-Tool. Second, the information regarding CO_2_-savings and monetary costs or savings indicated on the cards is individualized on the basis of the information given by the household in interaction 1. For example, the amount of money saved when room temperature is reduced is calculated for the size of the household (Fig. 3).

##### Procedure

Approximately two to 4 weeks after a household has filled in the online (or paper) questionnaire, Interaction 2 starts. Interaction 2 typically takes place in the household’s home, but may also take place in a neutral place if required by the interviewee.

**Fig. 4:**
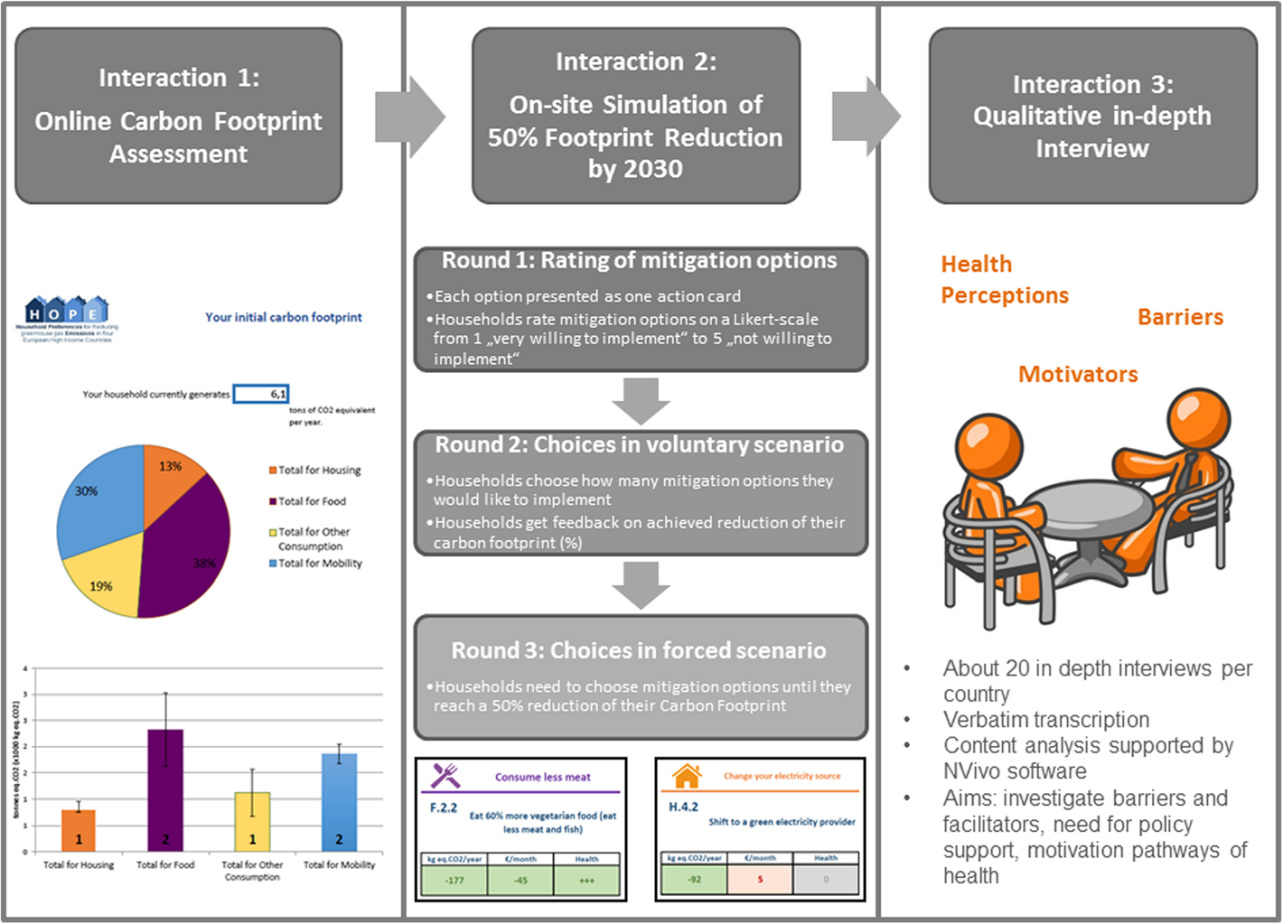
Tasks in the three rounds of the on-site simulation in Interaction 2

**Fig. 5:**
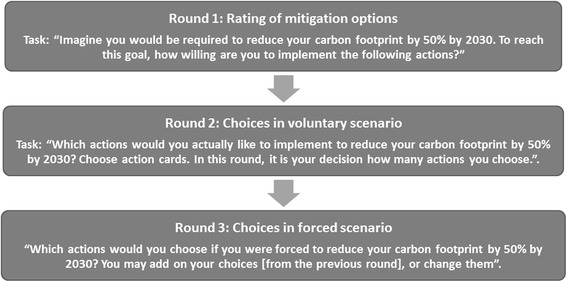
Overview on HOPE Study Protocol

At the beginning of the Interaction 2, the interviewer familiarizes the household representative with the concept of the carbon footprint, and informs about the household’s initial carbon footprint. The interviewer presents figures displaying the total amount of emissions (e.g. 17 tons CO2-e/year), and emission shares in the sectors housing, food, mobility, and other consumption in a graphic way (see Fig. 4, left side). The understandability of the figures has been tested in the pilot study and co-developed with a cognitive psychologist specialized in climate change communication. After this introduction, the on-site simulation starts. The interviewer explains to the representative that there is a target for households to reduce their carbon footprint by 50% by 2030, and asks the representative to approach this aim within three rounds (see Fig. 5).

In the first round (*rating of mitigation options*), the representative indicates his willingness to implement each measure on a Likert scale from 1 = very willing to 5 = not willing. To do so, the representative physically sorts each of the 65 action cards under one of five Likert scale labels indicating the values from 1 to 5 on the table. Rating scales are commonly used in psychological research to assess preferences. The assumption of an interval type level of measurement (equal intervals between the categories 1–5) is generally made to enhance statistical power (Norman, 2010).

In the second round (*Choosing and ranking in voluntary scenario*) the household representative chooses and ranks those actions that he or she would actually like to implement in his or her household. The interviewer enters the households’ choices into the FCS-tool that calculates the resulting reduction of the household. The representative is then shown graphs displaying (a) the reduced carbon footprint in total, (b) the initial and reduced carbon footprint for each of the four sectors housing, food, mobility and other consumption, and (c) a graph displaying the reduction that has been reached together with the 50% reduction target line. The point where representatives stop selecting measures can be seen as them implementing a red line, thus being unwilling to implement further measures.

In the third round (*choosing and ranking in forced scenario*), the household representative adds those measures he or she would most likely implement if they were forced to reduce their carbon footprint by 50% by 2030 (given the household did not reach a 50% reduction in round 2). While receiving continuous feedback on the current state of their carbon footprint reduction, the representative keeps selecting (less and less favorable) actions until the 50% emission reduction target is reached. The simulation is finished when the target is reached, the representative is unwilling to go further, or when there were no remaining solutions.

In a last step, the household representative fills out a follow-up questionnaire assessing perceptions of the simulation game and measuring variables formulated from concepts in the “Theory of Planned Behavior” [44], such as behavioral intentions and perceived control. The interviewer fills out a follow-up questionnaire assessing perceptions of the representative, and collecting topics that the household representatives talked about during the simulation game, especially but not exclusively pertaining to motivations and barriers for action, to inform the qualitative part of HOPE.

This information is collected in order to better assess the hypothetical and statement biases, which stated preferences surveys often generate. Individuals face significant difficulties in stating realistic preference without needing to commit to it, partly due to cognitive biases [45]. However, HOPE’s protocol is designed to lighten these limits, because the FCS-Tool creates plausible and individualized options for each household. Furthermore, the three-step approach in interaction 2 supports households in making informed decisions. This is because the rating of options in round 1 familiarizes the households with all options and allows for an informed choice in the voluntary and forced scenario of the simulation. Voluntarily choosing a sub-set of actions in round 2 allows households to make a realistic choice and stop as they reach a limit, which is the point after which they will not be willing to act. Thus, round 2 increases the incentive compatibility and consequentiality of the choice. When households choose additional actions in round 3 in order to reach the −50% reduction target, a compulsory dimension of choice is introduced. HOPE allows at various points to measure the level of construction of preferences and attitudes, which provides a strong signal of the level of decision for each household [46, 47].

#### Interaction 3: Qualitative in-depth interview

##### Scientific basis

Qualitative research enables researchers to understand experiences, attitudes or values that stand behind social phenomena [48]. In fact, qualitative research postulates that the perceived reality of individuals is socially constructed and therefore depends on personal experiences and beliefs [49, 50]. As these are very individual and hard to capture with structured quantitative research approaches, qualitative research applies other techniques to investigate social phenomena, such as open interviews or observations [51].

##### Procedure

Interaction 3 consists of qualitative in-depth interviews with a sub-sample of households that completed both Interactions 1 and 2. A semi-structured interview guide is developed based on the research questions, a literature review and preliminary results from interaction 1 and 2. The aim of the in-depth interviews is to explore households’ knowledge and perception of climate change and mitigation as well as their health perceptions in these areas. With regard to health perceptions, we will specifically explore the role of health co-benefits in participants’ decisions, including participants’ beliefs on how climate change will personally affect their individual or family members’ health, participants’ perceptions of which mitigation measures may be linked to health effects, or which specific health effects are perceived to be personally important.

An additional area of investigation will be the barriers, facilitators and dilemmas households encounter when reducing their carbon footprint. Within this area, interviews will explore perceptions of difficulty of reducing carbon footprints by 50% as well as perceived facilitators for making bigger lifestyle changes. The interview will also be used to investigate participants’ rationales behind their specific ways of prioritizing the mitigation options, and understand more about what kind of support households wish for to be able to make major reductions.

### Sampling and sample size calculation

Our sample consists of households from Bergen in Norway, the city of Communauté du Pays d’Aix in France, the city of Mannheim in Germany and the city of Umeå in Northern Sweden. Urban households are particularly vulnerable to some negative side effects of climate change but have, on the other hand, unique possibilities to take on a leading role in combating climate change [52]. Therefore, and to be able to engage closely with local stakeholders for research and dissemination we have focused on one city in each of the project countries.

#### The quantitative sample

The total sample size for quantitative Interactions 1 and 2 has been determined prior to data collection based on our hypothesis for the health research question. The hypothesis is that participants who are given the health information give better ratings to actions with positive effects on health than those participants who don’t receive the health information. We determined the sample size needed to test the experimental effect on the mean rating of mitigation measures (health information given versus no health information given) given one-sided testing and a small- to medium-sized effect of *d* = .3 (as found in framing studies, e.g. [31]), alpha = .05, and .8 power. This yields a total sample-size of *N* = 278.

#### The qualitative subsample

To obtain the subsample for the qualitative Interaction 3, we will apply the qualitative sampling technique of maximum variation sampling. Maximum variation sampling is a purposeful sampling that aims at investigating a diverse set of participants to cover a broad range of perspectives on the social phenomenon under study [53]. We are able to ensure the diversity of participants, as we will have socioeconomic data and results from interactions 1 and 2, which can inform our choice of participants for interaction 3. Data which we will use will be socio-economic criteria, such as age, gender, income, education or type of housing, but also information about the GHG footprint of the participant and his choices of mitigation options.

The definite sample size will be determined by the qualitative principle of saturation. Data saturation in qualitative studies is achieved, when the findings in newly won data sets, for instance interviews, reach a high grade of repetition without bringing up new themes [54].

### Recruitment

In a pilot study from December 2015 to January 2016 we sent out letters to a random sample of participants from city registries to test response rates. Since this technique yielded a rather low response rate of below 7%, we decided to proceed with a more open approach including a wider range of recruitment techniques such as advertisements in the local media as well as snowballing where necessary in the final study. In order to minimize selection bias, each participant completing Interaction 2 receives a voucher worth 25 Euro and the chance to win a prize worth a 1000 Euros in a scalable lottery. Scalable means that participants, who are open for participation in Interaction 3, increase their chance to win the prize worth 1000 Euros. This innovative form of lottery is particularly well-adapted to panel protocols with long interviews. The double reward can motivate different kind of participants (risk averse or risk seeking), and increase both the participation rate (voucher) and the completion rate (scalable lottery). Finally, this incentive scheme is efficient to reduce both the non-response bias and the measurement error [55, 56].

Due to the length and the intensity of the protocol (three interaction, one online and two in person meetings) and the available resources of the project we are also not able to increase the sample size to much over 100 participants per city. However, to maximize the generalizability of our results, given the restricted sample sizes, each country follows up the recruitment procedure with a guide of demographic criteria. These criteria include: type of housing (collective, individual), geographic location (central, urban, rural), presence of children (<18 years), state of household’s ownership (owner, renter), age group (18–35, 36–50, 51–65, 65+), and gender. In iterative recruitment rounds, the cells with respect to selected criteria can be filled in progressively. This guide is primarily used as a control function, to make sure that the recruited participants reflected each city’s population.

### Calculation of health effects

As the interaction 2 aims at assessing individual action preference, we only considered information on direct health co-benefits, which are accessible to household member as described in the introduction (see Fig. 1). We estimated the individual health impact of each mitigation measure in terms of quality adjusted life years (QALYs). QALYs are computed by examining the effect on age-specific mortality from the change in exposures or health-related behaviors associated with an estimate of the decrease in the quality of life for the additional years lived with a disease or disability. Quality weightings are represented on a 0–1 scale, with 1 representing full health. As an example, 3 years lived with quality rating 0.5 gives 1.5 QALYs. Dying a year early gives a loss of 1 QALY. Our estimates of loss in QALYs for each household measure are informed by the results of modelling studies of the health effects of low carbon interventions in areas as diverse as electricity production [18], housing [57], transport [58] and food and agriculture [59] were based on published detailed analyses for the UK, the results of which were then translated into semi-quantitative estimates by extrapolation to the study population. Because of uncertainties we classified the likely impact on life expectancy using four categories: −1 month, < +1 month (small effect), +1–3 months (moderate effect), and > +3 months (substantial effect).

Out of the total of up to 65 mitigation measures, 11 generate a positive health impact for the individual, one exerts a negative health impact, and 53 actions do not have a clearly proven health impact on the individual. Measures are not labeled as having a proven health co-benefit on the individual (even if they might be perceived as healthy; e.g., eating organic food), if:

(1) The health effect is very modest if only one person adopts a mitigation measure, and is only appreciable at population level (e.g., reduced air pollution);

(2) There is not enough scientific evidence to conclude about a health effect to date. This does not necessarily imply that a health effect does not exist; or.

(3) There is conflicting evidence or conflicting arguments (e.g., use of nuclear power is good for the climate and okay for health as long as there are no accidents). Please note that this constitutes a highly (and deliberately) conservative apprehension of health co-benefits. Table 1 shows mitigation options given to households in this study, which exert an established and scientifically proven health co-benefit as a direct effect (as depicted in Fig. 1). We want to point out that in this study we only consider health co-benefits, which are accessible to the individual by personal behavior change.

**Table 1:** Mitigation measures with health effects. Household mitigation measures exerting an established and scientifically proven health co-benefit on the individual, together with the strength and direction of the effect

Mitigation measure	Strength and direction of health effect (in QALYs)	Explanation
H.1.1 Insulate your roof/ attic.	<1 month +	Reduced cold-related health problems (including improved mental well-being), and lung and heart disease
H.1.2 Insulate your walls.	<1 month +	Reduced cold-related health problems (including improved mental well-being), and lung and heart disease
H.1.3 Improve your windows (increase glazing of your windows).	<1 month +	Reduced cold-related health problems (including improved mental well-being), and lung and heart disease
H.2.3 Lower in-house temperature by 3 °C	<1 month -	Some increase in risk of cold-related health problems if winter indoor temperatures fall below around 18 °C
F.2.1 Eat 30% more vegetarian food (less meat and fish).	> 3 months +++	Reduced risk of heart disease and some cancers
F.2.2 Eat 60% more vegetarian food (less meat and fish).	> 3 months +++	Reduced risk of heart disease and some cancers
F.2.3 Become a vegetarian (stop eating meat and fish).	> 3 months +++	Reduced risk of heart disease and some cancers
F.3.1Gradually give up on ready-made meals (e.g. frozen pizza, canned soups, frozen lasagne).	1–3 months ++	Reduced risk of stroke, heart disease and some cancers
M.1.1 Shift significantly (more than 30%) from car to public transport (bus, tramway, metro, train).	1–3 months ++	Reduced risk of heart disease, some cancers, diabetes, obesity and dementia; Neg: Increased in risk of road injury
M.1.2 Shift to non-motorized modes of transport (walk, bike) instead of public transport.	1–3 months ++	Reduced risk of heart disease, some cancers, diabetes, obesity and dementia; Neg: Increased in risk of road injury
M.2.2 Decrease your travels with cars public transport and other motorized vehicles by 30%.	1–3 months ++	Reduced risk of heart disease, some cancers, diabetes, obesity and dementia; Neg: Increased in risk of road injury
M.2.3 Give up your car(s) and other motorized vehicle(s)	1–3 months ++	Reduced risk of heart disease, some cancers, diabetes, obesity and dementia Neg: Increased in risk of road injury

### Data analysis plan

#### Mixed-methods analysis

The idea of an explanatory mixed-methods design is to explain the findings or remaining open questions form the quantitative part with the results of the qualitative part. This means that the results from the quantitative research should already be used in developing the qualitative research part. Thus, the quantitative part can inform the qualitative part to ask the right questions. This is one way how different methods can intertwine in mixed-methods research.

In addition to that, measuring the same social phenomenon with different research methods can alleviate measurement errors. If two imperfect tools (and measurement tools are never perfect) bring up the same findings, the confidence that can be put in those findings is higher. This principle is also known as across-method triangulation [60].

In our study we start with the analysis of the quantitative results in order to inform the development of the interview guide for the in-depth interviews. Combining the findings of households’ ratings and choices in Interactions 1 and 2 with the qualitative findings of related barriers, facilitators and health perception in Interaction 3 will enable us to gain a deeper understanding of health co-benefits as motivators for European households’ climate action. We will also strive to understand the rationales behind participants’ stated preferences in the quantitative part with the help of the qualitative results. Furthermore, any new themes, which may be identified via qualitative exploration, will be fed back into the quantitative analysis, if possible.

#### Quantitative data analysis

First, in the quantitative analysis, analyses of variance (ANOVAs) will compare households’ preferences to implement mitigation measures with vs. without the additional health information with respect to (1) the main quantitative dependent variable, which is the mean rating of the measures, and also (2) number of measures with positive health effect that were selected in the voluntary scenario, and (3) number of measures with positive health effect that were selected in the forced scenario. Second, a regression analysis will be performed to assess the strength of a possible effect of the health information on the dependent variables while controlling for a number of possible confounds (demographic variables and household characteristics). Independent variables collected in HOPE are listed in the Additional file 1. The experimental approach concerning the health information will allow to investigate a causal relationship if differences in-between groups are found. We expect to find that households receiving the additional information on health co-benefits (1) will rate measures with health co-benefits more positively compared to households not receiving health information; (2) will choose measures with health co-benefits more, and earlier than measures without health information (3), and will choose measures with health co-benefits more, and earlier compared to households not receiving health information.

Second, additional exploratory analyses will investigate for which *category* the hypothesized benefit of information on health co-benefit exists, or is particularly strong (i.e., food, housing, mobility, other consumption). Third, additional exploratory analyses will investigate for which *household representative* the hypothesized benefit of information on health co-benefit exists, or is particularly strong (e.g., those with children or women).

#### Qualitative data analysis

The in-depth interview will be recorded and transcribed verbatim. The transcripts will be analyzed with the method of content analysis supported by NVivo software. This is achieved by subsuming meaning units under a common classification in order to work out important themes and patterns in the data [61]. Because we want to openly explore study participants’ knowledge and perception on climate change mitigation and health we will analyze the in-depth interviews primarily inductively. This means that no predefined coding schemes are projected to the data, but that the codes and classifications will be grounded in the data.

#### Accompanying policy analysis

Within the transdisciplinary research team a policy analysis on household targeted climate policies in the four partner countries is conducted. There will be a special emphasis in the review about the health aspects considered in those policies. This will allow us to directly relate and compare our findings to current policies in order to develop relevant policy recommendations.

## Discussion

HOPE adopts a mixed-methods approach including elements of serious gaming, which is an innovative approach to investigate health co-benefits as a motivator to climate action. It is a strength of this protocol that serious gaming elements ensure the engagement of participants into the study. Furthermore, triangulation of different methods will allow the production of robust results.

On the other hand the time consuming study protocol doesn’t allow for sample sizes, which are representative for the country population. Yet, the sample sizes will be big enough to test for a causal effect of information on health co-benefits given or not given due to an experimental design. Additionally, the sample comprises four European high-income countries, which allows for international comparison.

The information on health co-benefits are directly accessible for households and provided for single climate mitigation measures (as opposed to general health framing in previous studies). Furthermore, the interdisciplinary assessment of households’ preferences for mitigation measures will have practical implications for public health, climate policies, and climate change communication. To ensure effective dissemination of results a policy advisory board with local, regional and national stakeholders from politics and society has been assembled in each project country from the start.

Finally, HOPE investigates whether health co-benefits can serve as a motivator for households to adopt mitigation measures. This is one of the first comprehensive mixed-methods approaches to this question that combines quantitative and qualitative methods with an experimental element. HOPE will deliver important input to the debate on how to tackle climate change: HOPE will help clarify which measures European households are most willing to implement in order to reach the 1,5° target set by the Paris Agreement. This will enable us to compare how these measures align with current policies in European high-income countries and provide knowledge for tailoring effective climate mitigation and health policies.

## Additional file

Additional file 1: List of variables collected in HOPE. (PDF 191 kb)

## Abbreviations

ANOVAs: ANalysis Of VAriance
COP: Conference of the Parties
EU: European Union
FCS-Tool: Footprint Calculation and Simulation Tool
GHG: Greenhouse Gas
IPCC: Intergovernmental Panel on Climate Change
UNFCCC: United Nations Framework Convention on Climate Change
WBGU: Wissenschaftlicher Beirat der Bundesregierung Globale Umweltveränderungen (German Advisory Council on Global Change).
