# Correction to: household preferences for reducing greenhouse gas emissions in four European high-income countries: does health information matter? A mixed-methods study protocol

**DOI:** 10.1186/s12889-017-4861-z

**Published:** 2017-10-25

**Authors:** Alina Herrmann, Helen Fischer, Dorothee Amelung, Dorian Litvine, Carlo Aall, Camilla Andersson, Marta Baltruszewicz, Carine Barbier, Sébastien Bruyère, Françoise Bénévise, Ghislain Dubois, Valérie R. Louis, Maria Nilsson, Karen Richardsen Moberg, Bore Sköld, Rainer Sauerborn

**Affiliations:** 1 Institute of Public Health, Heidelberg University Hospital, Im Neuenheimer Feld 130.3, 69120 Heidelberg, Germany; 2 Institute of Psychologys, Heidelberg University, Heidelberg, Germany; 3 TEC-Conseil, Marseille, France; 4 Vestlandforsking, Sogndal, Norway; 5 Department of Public Health and Clinical Medicine, Epidemiology and Global Health, Umeå University, Umeå, Sweden; 6 Centre International de Recherche sur l’Environnement et le Developpement (CIRED), Nogent, France

## Correction

After publication of the article [1], it has been brought to our attention that the titles of Figs. 4 and 5 have been transposed. Figure 4 should be titled “Overview on HOPE Study Protocol” and Fig. 5 should be titled “Tasks in the three rounds of the on-site simulation in Interaction 2”. The original article has been revised to reflect this.

**Fig. 5:**
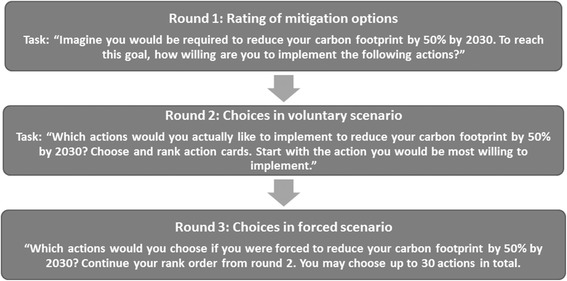
Tasks in the three rounds of the on-site simulation in Interaction 2

Following the publication of the erratum [2], it has been brought to our attention that the wrong version of Fig. 5 was included in the original article [1]. The correct version of the article is shown below.
