# Supplementary material for: Household preferences for reducing greenhouse gas emissions in four European high-income countries: Does health information matter? A mixed-methods study protocol
Source: BMC Public Health. 2017 Aug 1;18:71. doi: 10.1186/s12889-017-4604-1 (PMC5540303; doi:10.1186/s12889-017-4604-1)
Supplement: Additional file 1: — List of variables collected in HOPE. (PDF 191 kb) [file 12889_2017_4604_MOESM1_ESM.pdf]

Appendix. List of variables collected in HOPE.

N	Category name	Number of variables	Description
0	Interview	2	Interview information
1	Household data	458	HH data from Int1
2	Emission data	17	Computed CO2 emission data based on Int1 data
3	Cost data	2	Computed costs based on Int1 data
4	Likert ratings	65	Int2: rating of the 65 mitigation measures on a 5-point Likert scale
5	Ranking	136	Int2: ranking of mitigation measures
6	Timestamp	60	Int2: time stamp marking the selection of a mitigation measure (non-functional)
7	Already done actions	66	Action already done by household before survey
8	CO2 DATA (maximum potential)	70	Int2: Computed maximum potential of CO2 emission reduction (based on all 65 actions)
9	CO2 DATA when applicable	70	Int2: Computed maximum applicable CO2 emission reduction (based on applicable actions)
10	CO2 DATA Reduction Sim1	70	Int2: Computed CO2 emission reduction after simulation 1
11	CO2 DATA Reduction Sim2	70	Int2: Computed CO2 emission reduction after simulation 2
12	Cost DATA (maximum potential)	70	Int2: Computed maximum potential of cost change (based on all 65 actions)
13	Cost DATA when applicable	70	Int2: Computed applicable cost change (based on applicable actions)
14	Cost DATA Reduction Sim1	70	Int2: Computed cost reduction after simulation 1
15	Cost DATA Reduction Sim2	70	Int2: Computed cost reduction after simulation 2
16	Questionnaire: Interviewer	16	Feedback from interviewer after Int2
17	Questionnaire: Participant	15	Feedback from participant after Int2
18	TOOL VERSION	2	Excel tool version
	**Total**	**1399**	

Please note. Int1 = Interaction 1 - Online questionnaire; Int2 = Interaction 2 - On-site simulation.
